# Obturator Neurectomy for the Treatment of Adductor Spasticity: A Novel Technique and Case Series

**DOI:** 10.7759/cureus.74177

**Published:** 2024-11-21

**Authors:** Maximillian S Feygin, Michael Larkin, Dan Curry, Scott B Rosenfeld, Aloysia Schwabe, Nisha Gadgil

**Affiliations:** 1 Neurological Surgery, Baylor College of Medicine, Houston, USA; 2 Pediatric Neurosurgery, Texas Children's Hospital, Houston, USA; 3 Pediatric Orthopedic Surgery, Texas Children's Hospital, Houston, USA; 4 Physical Medicine and Rehabilitation, Texas Children's Hospital, Houston, USA

**Keywords:** cerebral palsy, management, neuromuscular hip dysplasia, obturator neurectomy, spasticity

## Abstract

Background

The management of adductor spasticity and long-term sequelae for cerebral palsy (CP) patients is complex. Hip displacement is a common consequence of CP, and obturator neurectomy (ON) is a potentially underutilized procedure to address the underlying adductor spasticity. The aim of this study is to describe the operational technique of ON and highlight the potential efficacy of ON in reducing spasticity, as well as pain, hip, and functional outcomes in these patients.

Methods

A total of eight patients from Texas Children’s Hospital who underwent ON between 2008 and 2023 were included in this case series.

Results

ON led to a qualitative decrease in adductor spasticity and had high patient-reported satisfaction. The average length of stay was 1.6 days (range: 1-4 days). Hip outcomes improved in all patients, evidenced by increased hip range of motion, improved mobility/gait, and decreased migration index (MI) in one patient.

Conclusions

ON is an efficient procedure that has the potential to reduce adductor tone and improve hip outcomes. The operative technique described and the reported patient satisfaction support the integration of ON into the paradigm of adductor spasticity management. Further prospective studies, however, are needed to objectively measure tone and hip outcomes in these patients.

## Introduction

Cerebral palsy (CP) encompasses a range of conditions in which an insult to the developing nervous system results in limitations of movement or posture [1]. Spasticity, defined as a velocity-dependent resistance of muscle stretch, often results and can affect up to two-thirds of children with CP [2]. Hip adductor muscles are commonly affected and can result in a narrow or scissoring gait that interferes with walking, transfers, and balance. In more severely affected children, it can create difficulty with perineal hygiene, dressing, positioning in wheelchairs, and other assistive devices [3-5]. The uncontrolled spasticity of adductor muscles can also contribute to pain and neuromuscular hip subluxation [3-5]. The management of spasticity requires a multidisciplinary team, and early interventions are recommended to prevent long-term sequelae [2,3,6-7]. There are many options, both medical and surgical, to manage spasticity that typically follow an escalating paradigm [2,3,6,8]. Management strategies operate along two lines of approach, addressing systemic or focal tone and being either reversible or permanent [9,10]. Oral baclofen provides systemic, reversible tone reduction and is generally the first line for spasticity [1,3,8]. In conjunction with Baclofen, chemodenervation with botulinum toxin injection (Botox) or phenol injections can provide reversible focal reduction [1,3,8]. Permanent strategies include intrathecal baclofen pumps (ITB) or selective dorsal rhizotomies (SDR), which provide systemic or focal reductions, respectively, and are reserved for more refractory cases of spasticity [1,11,12]. An ITB is a microinfusion device that provides a continuous and titratable infusion of Baclofen intrathecally [10,12]. Though considered quite effective in tone reduction, ITB pumps have significant drawbacks, such as routine maintenance/refills, infection and wound healing complications, and the risk of baclofen withdrawal due to pump malfunction [12]. SDR is a permanent tone-reducing strategy in which dorsal nerve roots are selectively sectioned in the lumbosacral spine (L2 to S1), thereby reducing input to the muscle stretch reflex underlying spasticity [10,11]. Candidacy for this surgery is limited to children who are ambulatory, young, have predominant spasticity, have a good level of cognition and participation in therapies, and have minimal existing musculoskeletal deformity [11].

Neuromuscular hip subluxation is a prevalent long-term sequelae seen in up to one-third of patients with CP [13-15]. Previous studies have reported a 68-90% risk for subluxation and progressive displacement in non-ambulatory children [13,16]. Prior studies have investigated the role of spasticity management using Botox [17-19], ITB [20,21], or SDR [22] in slowing neuromuscular hip subluxation but have demonstrated mixed results. Two studies investigating chemodenervation with Botox, by Yang et al. and Graham et al., found a statistically significant effect in reducing spasticity in children with bilateral spastic CP [17,18]. A follow-up to Graham's study measuring hip subluxation an average of 10 years later, however, concluded that long-term subluxation and hip morphology were not affected [19]. One study looking at non-ambulatory patients with ITB found no significant difference in the rate of hip subluxation or rates of surgeries compared to patients without ITB over five years [20]. This result opposes a shorter-term study, one year post-implantation, which demonstrated a significant reduction in the rate of change in hip subluxation in 90% of patients [21]. Investigating SDR, a study by Dudley et al. evaluating the long-term benefits of the procedure found that ambulatory patients with CP who undergo SDR before the age of five can protect against declining functionality in adolescence and progressive hip dislocation [22].

Selective anterior obturator neurectomy (ON) is an underutilized procedure, described by Sindou et al., that can provide permanent, focal tone reduction by isolating and selectively sectioning the anterior branch of the obturator nerve [9]. While the literature is sparse, a study by Ren et al. showed potential use for ON in managing neuromuscular hip subluxation [23]. In this paper, we describe our experience with a cohort of patients undergoing ON, highlighting the potential to improve tone and hip subluxation, describe the technique in detail, and provide clinical pearls and potential indications for the appropriate use of this tool in treating adductor spasticity.

## Materials and methods

Obturator neurotomy surgical procedure

**Figure 1:**
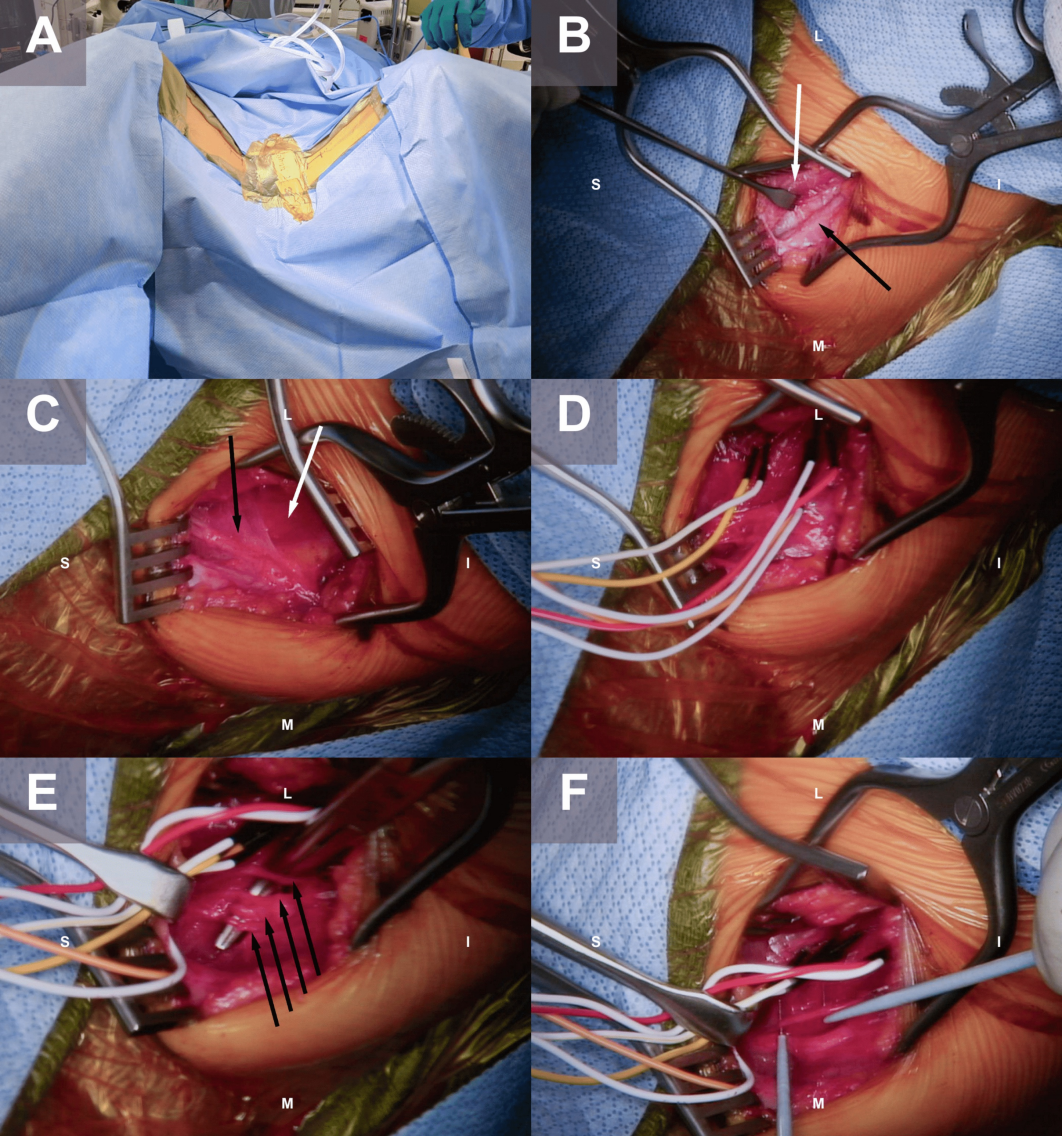
Intraoperative photographs of a patient undergoing bilateral obturator neurectomy A) The patient is in the lithotomy position in stirrups. Vertical incisions are marked 2 cm below the hip crease along the adductor tendon, approximately 3 cm in length. B) Close-up of the left-sided incision is subsequently pictured. Following subcutaneous dissection, the adductor muscles are encountered. The plane between the gracilis muscle (medial, black arrow) and adductor longus (lateral, black arrow) is dissected. C) The adductor longus and gracilis have been retracted, exposing the underlying adductor brevis muscle (white arrow). The anterior branch of the obturator nerve is found coursing over the adductor brevis (black arrow). D) Sterile electromyography needles are inserted into the adductor brevis (yellow), adductor longus (red), and adductor magnus (orange, deep) muscles. E) Branches of the anterior obturator nerve to the adductor brevis, adductor longus, and gracilis are separated and dissected. F) Each branch is stimulated with rhizotomy hooks until a compound muscle action potential (CMAP) is obtained, then selective sectioning is performed

General anesthesia is induced, avoiding the use of long-acting paralytics that interfere with intraoperative electrophysiology. Antibiotic prophylaxis is given. The patient is positioned in the lithotomy position in stirrups, exposing the medial thigh bilaterally (Figure 1A). A vertical incision is marked 2 cm inferior from the hip crease along the adductor tendon, about 3 cm in length. The incision is infiltrated with local anesthetic and incised. Dissection continues through subcutaneous tissues until the adductor muscles are identified. The most superficial muscle exposed is the adductor longus muscle, and just medial to it is the gracilis. The adductor longus is mobilized and retracted laterally, and the gracilis is retracted medially (Figure 1B), exposing the underlying adductor brevis muscle. The anterior branch of the obturator nerve is found coursing over the adductor brevis (Figure 1C). Sterile electromyography needles are inserted into the adductor brevis, adductor longus, and adductor magnus (deep to adductor brevis) muscles (Figure 1D). Branches of the anterior obturator nerve to the adductor brevis, adductor longus, and gracilis are identified and dissected (Figure 1E). These are sequentially stimulated with rhizotomy hooks until a compound muscle action potential (CMAP) is obtained (Figure 1F). Stimulation of nerve branches arising from the anterior branch of the obturator nerve should produce CMAPs of the adductor brevis, longus, and gracilis, but not the adductor magnus. We intraoperatively evaluate the CMAP response in target muscles. About 50-100% of the branches are sectioned, which is operator-dependent and also dependent on the extent of preoperative adductor spasticity. The identical procedure may be performed contralaterally.

Case series

Patients were screened from the Texas Children’s Hospital neurosurgical database from the years 2008 to 2023 who have undergone selective anterior ON by CPT code (64766). A comprehensive retrospective chart review was performed to extract all the relevant data on each patient’s obturator neurotomy procedure and related outcomes.

Data extracted included demographic information such as gender and age at surgery, as well as information about an underlying diagnosis, Gross Motor Function Classification System (GMFCS) [24], and adductor Modified Ashworth Scale (MAS) (if available). The MAS score is used to measure increased tone on a scale from 0 to 4; 0 indicates no increase in muscle tone, and 4 indicates muscle rigidity in flexion or extension [25]. GMFCS and MAS were reported by patients’ treating pediatric physical medicine and rehabilitation (PM&R) providers in the clinic or by their physical therapists. Additionally, past medical and surgical history, including information about current medications for tone, prior Botox or phenol injections into the adductors, and any prior orthopedic or neurosurgical procedures, were recorded. Preoperative information was collected, including the rationale for surgery, preoperative hip position, migration index (MI) (if available), ambulatory status, and gait analysis (if available). Operative information, including the number of branches cut, concurrent procedures performed, length of stay, and postoperative complications, was also collected. Last, data on the reported outcomes were extracted for reported patient/family satisfaction, pain improvement, effect on tone, ambulation, gait, hip (MI if reported), and whether further Botox injections or adductor lengthening procedures were needed.

The primary endpoint of the study was adductor MAS. Secondary endpoints included improved “hip outcomes” defined as either a qualitative or quantitative improvement in range of motion (ROM), “tone outcomes” defined as a decrease in tone noted during the physical exam, a reported decrease in the spasticity of adductors, or a decrease in MAS. These endpoints were measured at six months and one year. The need for further Botox injections or adductor lengthening procedures was another secondary outcome.

## Results

Eight patients who underwent ON were identified and retrospectively reviewed. All patients had an underlying diagnosis of CP and clinical manifestation of mixed tone with both dystonia and spasticity. The average age at surgery was 17 years old (range: 7-24), and two out of eight patients were considered ambulatory (GMFCS 3). The average GMFCS score for patients was 4 (range: 3-5). All patients were on oral baclofen for tone management, except one patient in whom it was discontinued due to side effects. Prior procedures included Botox or phenol injections to control for tone in leg muscles (typically adductors, hamstrings, and gastrocnemius). All patients received chemodenervation injections prior to ON except patient 5. This patient suffered respiratory distress after an attempted injection. For all other patients, injections were performed at variable time intervals. Table 1 describes the specific patient details.

**Table 1:** Patient information * indicates patient trialed baclofen, however, discontinued it due to side effects; ** indicates patient developed respiratory distress following Botox injection CP: cerebral palsy; GMFCS :Gross Motor Function Classification System

Case number	Gender	Age at surgery	Diagnosis	Ambulatory (yes/no)	GMFCS	Takes baclofen for tone	Prior Botox/phenol to adductors	Tone improvement with Botox
1	M	19	Diplegic CP	Yes	3	Yes	Yes	Yes
2	F	18	Dystonic quadriplegic CP	No	5	Yes	Yes	Yes
3	M	17	Spastic diplegic CP	No	4	Yes	Yes	Yes
4	M	19	Spastic triplegic CP	No	4	Yes	Yes	Yes
5	M	7	Spastic quadriplegic CP	No	5	Yes	No**	N/A
6	F	24	Diplegic CP	No	4	Yes	Yes	Yes
7	F	21	Diplegic CP	No	4	Yes	Yes	Yes
8	M	12	Spastic quadriplegic CP	Yes	3	No*	Yes	Yes

ON was performed by dissection and selective sectioning of at least 50% of the anterior branch of the obturator nerve bilaterally. The average percentage of branches cut was 74% (range: 50%-100%). Adductor tenotomy was performed concurrently in two patients via the same incision. The average length of stay for the procedure was 1.62 days (range: 1-4). All except one patient were discharged home within four days. One patient required inpatient rehabilitation post-surgery. This patient, who underwent tenotomy and ON, experienced a decline in his functional baseline and required three weeks of inpatient rehabilitation. Table 2 outlines the salient details of the ON procedure.

**Table 2:** Efficacy of obturator neurectomy * indicates adductor tenotomy performed concurrently; ** indicates patient declined from functional baseline requiring assistance with activities of daily living (dressing, showering) and mobility due to deficits in transfers, spasticity, lower limb weakness, and neuropathic pain

Case number	Branches cut	Concurrent procedures	Length of stay (days)	Postoperative complications	Further Botox needed
1	2 of 4 (50%) bilaterally	No	4	No	No
2	5 of 5 (100%) bilaterally	Yes*	2	No	Yes
3	5 of 5 (100%) left 3 of 5 (60%) right	Yes*	2	Yes**	No
4	5 of 7 (71%) left 6 of 8 (75%) right	No	<1	No	Yes
5	4 of 5 (80%) bilaterally	No	<1	No	No
6	5 of 6 (83%) left 4 of 5 (80%) right	No	<1	No	No
7	1 of 2 (50%) bilaterally	No	1	No	No
8	4 of 5 (80%) bilaterally	No	1	No	No

The primary outcome was a decrease in adductor MAS; this data was only recorded in half the cohort and was improved by an average of 1.25 (SD: 0.83). In the remaining patients, the MAS was not recorded, though the physician-reported tone was noted to be qualitatively improved on the physical exam. Improvements in patient- or caregiver-reported outcomes included improved ease of perineal hygiene/diapering, improvement of ambulation/gait, and pain reduction, with six out of eight patients reporting that they were newly pain-free. Table 3 reports the relevant primary and secondary outcomes. This also includes “hip outcomes,” which include an improvement in ROM, no change in MI, or a decrease in MI. The average preoperative MI was 24% (range: 2%-68%). Patient 5 was the only patient to receive pre- and post-MIs, demonstrating a reduction from 68% to 59%, nine months following ON. Further Botox injections needed after surgery were also reported, with two out of eight patients needing further injections into their adductors (Table 3).

**Table 3:** Obturator neurectomy effects on pain, tone, and hip outcomes * indicates that decreased scissoring was reported; however, there was a significant increase in tone in hamstrings and gastrocnemius NR: not reported; MAS: modified Ashworth scale; MI: migration index

Case number	Preoperative adductor MAS	Postoperative adductor MAS	Preoperative MI	Postoperative MI	Pain improvement?	Improved tone in six months?	Improved hip outcomes in six months?	Improved tone in one year?	Improved hip outcomes in one year?
1	2	NR	19%	NR	Yes	Yes	Yes	No*	No*
2	4	3	29%	NR	Yes	Yes	Yes	NR	NR
3	3	1+	20%	NR	Yes	Yes	Yes	Yes	Yes
4	1+	1	4%	NR	Yes	Yes	Yes	No	No
5	NR	NR	68%	59%	NR	Yes	Yes	NR	NR
6	3	1+	33%	NR	Yes	Yes	Yes	NR	NR
7	2	NR	18%	NR	Yes	Yes	Yes	NR	NR
8	2	NR	43%	NR	Yes	Yes	Yes	NR	NR

## Discussion

Selective peripheral neurectomy is an overlooked but potentially powerful tool in the arsenal of the neurosurgeon evaluating patients with hypertonia. ON affects focal, permanent tone reduction in the adductors of the hip. In this paper, we describe the technique in detail to address issues related to consistency and report outcomes in eight patients who have undergone this procedure spanning 15 years at a high-volume pediatric hospital. It is likely that this procedure is underused at our institution. There are several clinical pearls we would like to emphasize regarding this procedure.

By targeting the final common motor input at the muscle level, this procedure is helpful in reducing hypertonia related to either spasticity or dystonia. Preservation of the posterior branch of the obturator nerve, which innervates the adductor magnus, is essential in preserving the stabilization of the hip joint. Sectioning of the posterior branch may result in severe weakness in hip adduction and relative overactivity of the hip abductors, resulting in the dreaded hip abduction deformity. This complication may result in worsening movement and a broad-based gait, with resultant difficulty sitting and positioning in a wheelchair [26,27]. The use of neuromonitoring is necessary for this reason, and the “selective” nature of the procedure refers to the sectioning of the most reactive and therefore hypertonia-supporting nerve branches.

Botox injections are a safe, effective, and efficient treatment for spasticity that will predictably decrease tone for three to six months [28]. Preoperative chemodenervation, either with Botox injections or, more fittingly, phenol injections, is an essential component of the pre-surgical workup. These procedures mimic, temporarily, a similar effect as ON, and therefore serve as a helpful “trial” for the physician and caregiver to understand the utility of the procedure.

We suggest the following guidelines in evaluating the usefulness of ON for a patient with elevated tone. The focally impairing tone in the hip adductors interferes with gait (scissoring, leg crossing), care (difficulty with diaper changes or perineal hygiene), or pain. Adductor contracture with progressive hip subluxation may be a relative indication to consider the procedure; at this time, enough data does not exist to determine whether ON improves these outcomes. The procedure is likely to be more successful in preventing decline and the need for further surgery if the hip subluxation is not already severe [29,30]. We have also applied this procedure to patients with generalized lower extremity hypertonia in situations when the child 1) was not a candidate for global tone reduction, or 2) focal adductor tone reduction was felt to be beneficial while avoiding reduction of tone in useful, anti-gravity muscles, or 3) when the morbidity of an open lumbosacral rhizotomy could be avoided in the adductor hypertonia dominant patient. In addition to the above indications, the integration of ON into the management of adductor spasticity is reasonable when Botox and baclofen have decreased effectiveness and focal tone management is needed.

Results from this case series support the feasibility of this procedure and its potential efficacy in adductor tone reduction and neuromuscular hip subluxation. There was high self-reported patient satisfaction with the procedure, and the duration of hospital stay was low (mean: 1.5 days); same-day discharge is possible in many cases, and intensive postoperative rehabilitation is rarely required for this procedure. Rehabilitation and physical therapy are performed on an individualized basis, typically resuming after a period of 1-2 weeks after surgery on the patient’s previous schedule; however, the goals of physical therapy are more limited given the palliative nature of this surgery. Two patients (25%) required further tone reduction to their adductors through Botox/phenol injections. Residual tone may have been present due to underdosing of the neurectomy, missed branches of the anterior obturator nerve, or via the posterior branch of the obturator nerve that partially innervates adductor brevis and adductor Magnus. While we typically do not intraoperatively identify the posterior branch or stimulate it and do not advocate for sectioning of branches of the posterior branch, intraoperative identification and stimulation of all branches of the anterior nerve may mitigate this problem.

There are many limitations to this case series, including missing data and inconsistency in reported outcomes, with only half of patients with recorded MAS scores. The sample size of this single-center, retrospective series is low, highlighting the infrequency and perhaps underutilization of this procedure. Two out of eight patients underwent concurrent adductor tenotomies, which is a potential confound; however, this procedure should not affect postoperative tone. Additionally, the procedure is operator-dependent, affecting the consistency and reliability of outcomes. Further investigation into the role of ON in the treatment of adductor spasticity and hip subluxation is warranted to evaluate its possible protective role in neuromuscular hip displacement.

## Conclusions

The results from this case series demonstrate the potential for ON to be integrated as part of the treatment paradigm for spasticity. This article additionally highlights the technical approaches necessary to perform the procedure and specific indications for this patient population. This includes sectioning of at least 50% of the anterior obturator nerve, dependent on the extent of preoperative adductor spasticity and indications such as focally impairing tone to adductor muscles with the need for avoidance of global tone reduction or as a next step if Botox injections and ITB show reduced effectiveness.

Selective peripheral neurectomy, including ON, is just one of several tools in the neurosurgeon’s arsenal when evaluating a child with spastic CP. A multi-disciplinary approach, including PM&R, orthopedic surgeons, neurologists, and therapists, is key in recommending the optimal modality of medical and/or surgical treatment. A meticulous understanding of the patient’s functional deficits, the status of hip health and soft tissue deformity, and patients’ or caregivers’ concerns is essential.
